# Color-discrimination threshold determination using pseudoisochromatic test plates

**DOI:** 10.3389/fpsyg.2014.01376

**Published:** 2014-11-27

**Authors:** Kaiva Jurasevska, Maris Ozolinsh, Sergejs Fomins, Ausma Gutmane, Brigita Zutere, Anete Pausus, Varis Karitans

**Affiliations:** ^1^Department of Optometry and Vision Science, Faculty of Physics and Mathematics, University of LatviaRiga, Latvia; ^2^Institute of Solid State Physics, University of LatviaRiga, Latvia; ^3^Faculty of Biology, University of LatviaRiga, Latvia

**Keywords:** color vision, psychophysics, vision system, color measurement, color-vision deficiency, color-vision defects, color perception

## Abstract

We produced a set of pseudoisochromatic plates for determining individual color-difference thresholds to assess test performance and test properties, and analyzed the results. We report a high test validity and classification ability for the deficiency type and severity level [comparable to that of the fourth edition of the Hardy–Rand–Rittler (HRR) test]. We discuss changes of the acceptable chromatic shifts from the protan and deutan confusion lines along the CIE *xy* diagram, and the high correlation of individual color-difference thresholds and the red–green discrimination index. Color vision was tested using an Oculus HMC anomaloscope, a Farnsworth D15, and an HRR test on 273 schoolchildren, and 57 other subjects with previously diagnosed red–green color-vision deficiency.

## INTRODUCTION

Pseudoisochromatic (PIC) test plates are widely used for color-vision screening, congenital deficiency classification, and grading. Regardless of the design used in the creation of the PIC tests, they produce different grouping responses in people with normal trichromatic vision and abnormal color vision. Vanishing design PIC test plates are the easiest to produce, and all PIC tests contain these plates (Birch, 1993).

It is possible to obtain PIC tests with high performance (sensitivity and specificity approaching values of 1.00) by carefully choosing colored symbol chromatic values (Cole et al., 2006; Cole, 2007). PIC tests usually classify color-vision deficiency into three groups in terms of severity: mild, medium (moderate), and strong (severe). The total color difference (Δ*E*^∗^*_ab_*) of the plate stimuli determines the level of difficulty of the plate. For red–green color-vision deficiencies, the advisable values of Δ*E*^∗^*_ab_* measured according to the *Commission internationale de l’eclairage* (CIE) LAB formula are as follows:

• 15–22 units for small deficiency (screening),
• 30–40 units for medium deficiency, and
• 50–60 units for severe deficiency detection (Birch, 1993).

The gold standard for color-vision diagnostics is the anomaloscope testing procedure, which also characterizes the abnormality by assigning a quantitative value to the examination result. Other frequently used tests are usually based on error counting, which is further associated with the severity of color-vision deficiency based on measurement of chromatic detection thresholds. Although the anomaloscope is a precise instrument, it has been reported that the correlation between matching range results and the ability to perform everyday color-discrimination tasks, such as judging surface colors, is poor (Birch, 2008; Baraas et al., 2010). Nevertheless, the anomaloscope matching range of an individual is often used as a measure of the severity of the color-vision deficiency (Birch, 1993).

It would be convenient to characterize individual color-discrimination sensitivity in terms of a threshold value, such as the total color-difference value in the CIE LAB color space, for colors on confusion lines to perceive a noticeable difference. It has been suggested that scoring techniques for expressing test results in terms of sensitivity loss may be informative (Smith et al., 1993). We have developed a set of PIC plates for determining individual Δ*E*^∗^*_ab_* (hereafter abbreviated as Δ*E* for brevity) threshold values (Luse et al., 2012, 2013). Three main points distinguish KAMS (a term comprising the names of the test developers) from other PIC tests currently used in clinics. First, it is based on matching experimental data for color deficient individuals. Second, it characterizes the severity of deficiency in numerical units associated to sensitivity loss along the color confusion line. Third, the KAMS test is psychophysical, whereas other PIC tests, including Hardy–Rand–Rittler (HRR) and Ishihara, are not. The aim of this study is to assess the clinical performance and physical properties of our test plate set and to report its scientific relevance.

## METHODS

### DEVELOPMENT OF TEST PLATES

The KAMS test was designed using the principles described by Birch (1993), using neutral colors (i.e., colors that are confused with gray). In our previous work, we chose the chromaticities used for the KAMS plates. Five color-vision-deficient individuals (3 deutan, 2 protan) performed matching experiments under controlled illumination conditions (using a Qualitest CT-100W1 light booth under D65 illumination with a color temperature of *T* = 6500 K). In total, we created more than 300 chromatic and 106 gray color samples along and next to the deutan and protan color confusion lines passing through the achromatic area in the CIE *xy* color diagram. Samples had various saturation and lightness levels, and were 3 cm × 3 cm. The subjects were given two tasks: first, to sort samples into stacks of gray and chromatic, and second, to sort gray samples in piles of similar lightness. In a similar manner, via two subsequent trials subjects matched more samples, which we created, to achieve a gradual increase in Δ*E* in the KAMS test plates. A more detailed explanation can be found in our previous work (Luse et al., 2012).

**FIGURE 1:**
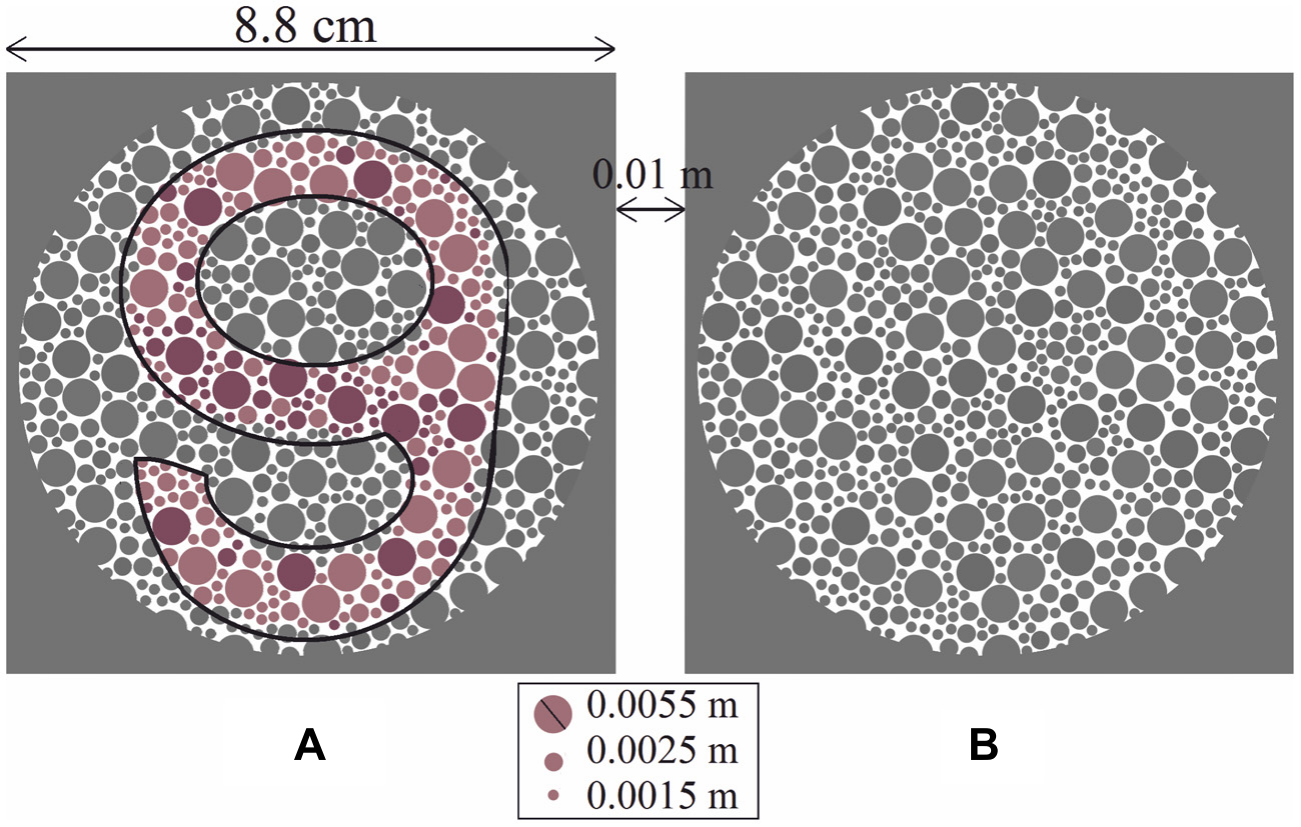
**Each large circle set (A) and (B) was composed of equal numbers of small dots, with three different sizes, some of which formed the shape of a symbol.** The proportion of lighter symbol-forming dots to darker symbol-forming dots was constant for all plates. Circle **(A)** contains the chromatic symbol “9” (shown with a black outline for clarity in the black and white prints of the manuscript), and circle **(B)** is empty. In this case, the deutan classification plate with Δ*E* = 27 is shown.

A set of 24 PIC plates [10 for protan deficiency (5 reddish and 5 greenish) and 14 for deutan deficiency (9 reddish and 5 greenish)] was created based on psychophysical design. Each plate had two circle sets (A) and (B), where only one held a chromatic symbol. The symbols used were four different digits with a rounded shape (i.e., 6, 8, 9, and 0), giving a guessing rate for each plate of 12.5%. **Figure 1** shows an example of a plate for detecting deutan deficiency (the corresponding Δ*E* for the printed plate is 27 units). The observers had two tasks: detection (to determine whether circle (A) or (B) contains a symbol) and recognition (to determine which of the four numbers the circle contains). Each plate was printed on a single page. The pages were turned at 3-s intervals.

The tests were printed using a calibrated inkjet printer (EpsonStylus Pro 7800) using nine UltraCrome K3 Epson Ink cartridges (four types of black, light cyan, light magenta, cyan, magenta, and yellow) on Premium Semimatte photopaper 260, with a resolution of 2880 dpi. It has been reported that higher quality can be achieved in terms of narrow chromatic value dispersion along the CIE *xy* color diagram by using inkjet printing (Luse et al., 2012, 2013) instead of the typography method used in the production of commercially available PIC plates (Lee and Honson, 2003; Dain, 2004; Fomins and Ozolinsh, 2011).

Increasing difficulty of the plates was achieved by decreasing Δ*E* of the test stimuli and the background. Such an approach has been reported previously (Wenzel and Samu, 2012). The symbol on test plates was composed of two stimuli–background-forming dot pairs (one lighter and one darker). The lighter pairs (making up 57% of the body of the symbol) were chosen to closely match the intensity (and accordingly the Δ*E*) of the darker ones; the deviation in most cases was less than a few units. The calculation of the color pair Δ*E* values was performed based on data obtained using a Konica Minolta CS-100A colorimeter (CIE *xy* diagram color space coordinate *x*, *y,* and *Y* values were recorded). Samples were illuminated with simulated D65 (*T* = 6500 K) illumination via a Qualitest CT-100W1 light booth. Color differences were calculated using the formula proposed by Berns (2006), taking into account the differences in sample lightness (Δ*L*^∗^), chroma (Δ*C*^∗^*_ab_*), and hue (Δ*H*^∗^*_ab_*), i.e.,

$${\Delta E}_{ab}^{*}=\sqrt{{\left({\Delta L}^{*}\right)}^{2}+{\left({\Delta C}_{ab}^{*}\right)}^{2}+{\left({\Delta H}_{ab}^{*}\right)}^{2}}.$$

### ANALYSIS OF PLATE COLOR STABILITY

**FIGURE 2:**
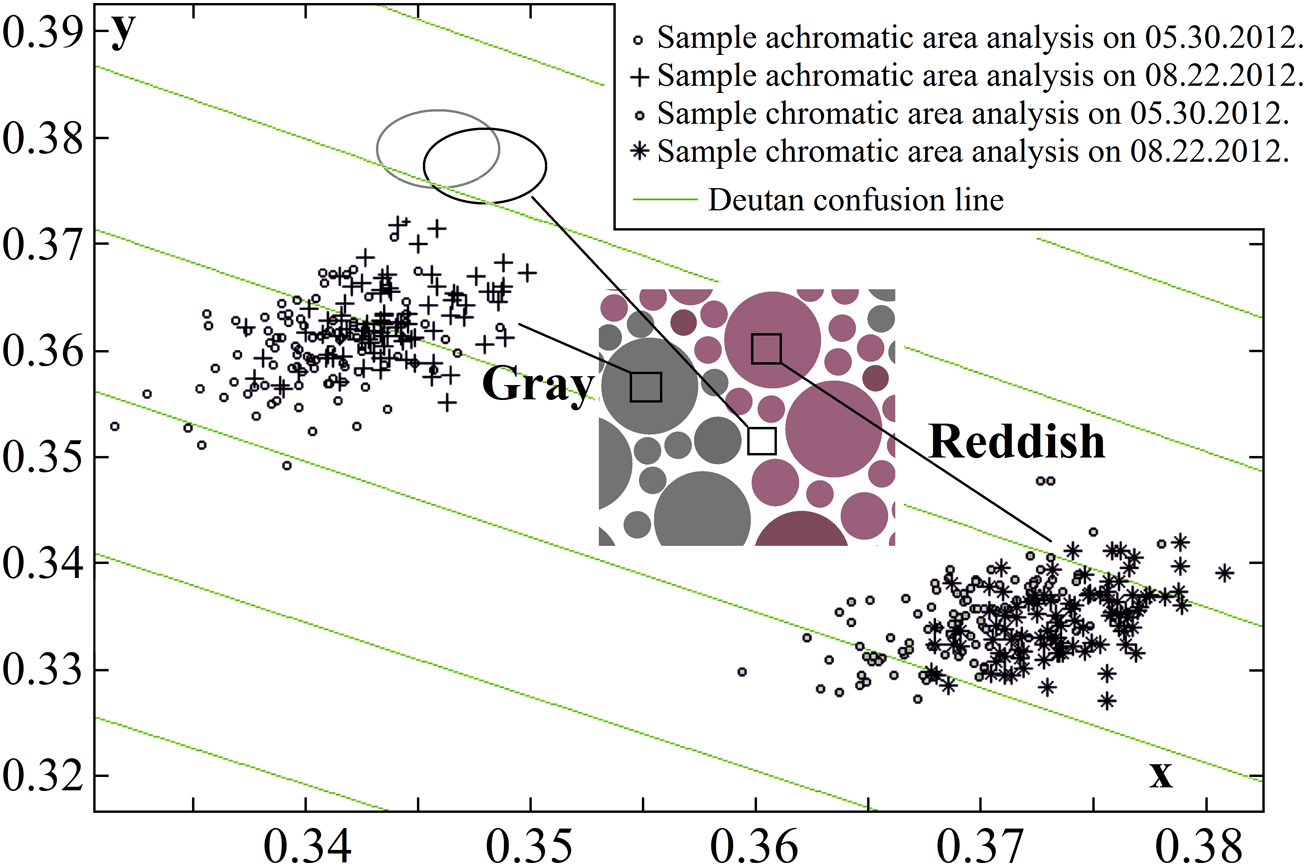
**Pseudoisochromatic (PIC) plate image pixel color dispersion changes over 3 months in the CIE *xy* color diagram (one plate stimulus is shown, and the corresponding plate is described in Figure 1).** Statistically significant fading was not detected (data points in both sets for both color pairs – chromatic and achromatic –overlapped and were of equal size). The ellipses show an analysis of the yellowing of the white paper (black ellipse to gray ellipse). The paper color coordinate values were more consistent than those of the printed areas, therefore the ellipses (and data point distributions that are not shown in the graph) were smaller than the other pixel value distributions.

Using calibrated inkjet printing instead of typography methods to produce PIC plates showed that several aspects should be considered, such as a smaller spread of chromatic values (Luse et al., 2012); however, the main drawback is faster changes in color over time (Lee and Honson, 2003; Lee, 2006). We have reported significant changes in the plate stimuli color saturation after 7 months of use (Luse et al., 2013). Prior to and throughout the study, the fading of pigments was monitored monthly using multispectral imaging, and the colorimeter measurements described above. Multispectral imaging was performed using a CRI Nuance Vis 07 multispectral camera with a Nikon AF-S Micro-Nikkor 60-mm *f*/2.8*D* objective lens. Image acquisition was performed in an otherwise dark room; the samples were inserted in the Qualitest CT-100W1 light booth; the samples were attached to the wall at a constant height and 50 ± 5 cm away from the camera, and were illuminated from above using a standard D65 light source (*T* = 6500 K). 1290 × 920 pixel spatial images were captured at visible wavelengths (420–720 nm, in steps of 10 nm). The images were transformed into cone excitation images using cone sensitivities [the method is described in detail in (Fomins and Ozolinsh, 2011)]. Selected image areas of 89 pixels were analyzed, and CIE *xy* color space (*x*, *y*, *Y*) values were acquired for each pixel. Changes in the mean color values of the pixels and the corresponding pixel distributions were not statistically significant during the first 3 months of use, as shown in **Figure 2**. The study described below was conducted over a period of 3 months using a newly printed, chromatically identical set of test plates. The mean standard deviation of the pixel value dispersion was 0.002–0.0028 units along the *x*-axis and 0.0026–0.003 along the *y*-axis (larger for darker samples).

### ANALYSIS OF SAMPLE RELATION TO COLOR CONFUSION LINES

Color pairs for the PIC plates were created via matching experiments carried out on several color-vision-deficient subjects (the experimental conditions are described in detail in Luse et al., 2012). Over the course of several trials, using several hundred samples, 60 valid color pairs (a reddish or greenish sample for stimuli and a corresponding gray sample for background) were obtained for construction of PIC plates. The colored dots on each plate had chromaticity coordinates that lie on or close to the protan or deutan confusion line connecting the dichromatic confusion line copunctal point and each chromaticity coordinate “cloud” center of the gray background color. The confocal points we used are given by CIE chromaticity coordinates (0.75, 0.25) for protan and (1.40, -0.40) for deutan deficiencies (Birch, 1993). All obtained chromatic sample relations (that is, the distances *d*) to confusion lines were analyzed to answer the following question: how far from the corresponding theoretical confusion line (for the congenital color-vision-deficient individual) may the colored stimuli chromaticity coordinate distribution center be situated and still prove to be indistinguishable from its achromatic pair? For these calculations, geometrical equations were used. If a line is given by two points (*x*_1_, *y*_1_; i.e., the deutan or protan confusion line copunctal point) and (*x*_2_, *y*_2_; i.e., the center of each achromatic sample pixel color coordinate distribution center), and the point in question is (*x*_0_, *y*_0_; i.e., the chromatic sample color coordinate distribution center), *d* can be found as follows:

$$d=\left|(x_{2}-x_{1})(y_{1}-y_{0})-(x_{1}-x_{0})\left(y_{2}-y_{1}\right)\right|÷\sqrt{{\left(x_{2}-x_{1}\right)}^{2}{\left(y_{2}-y_{1}\right)}^{2}.}$$

**FIGURE 3:**
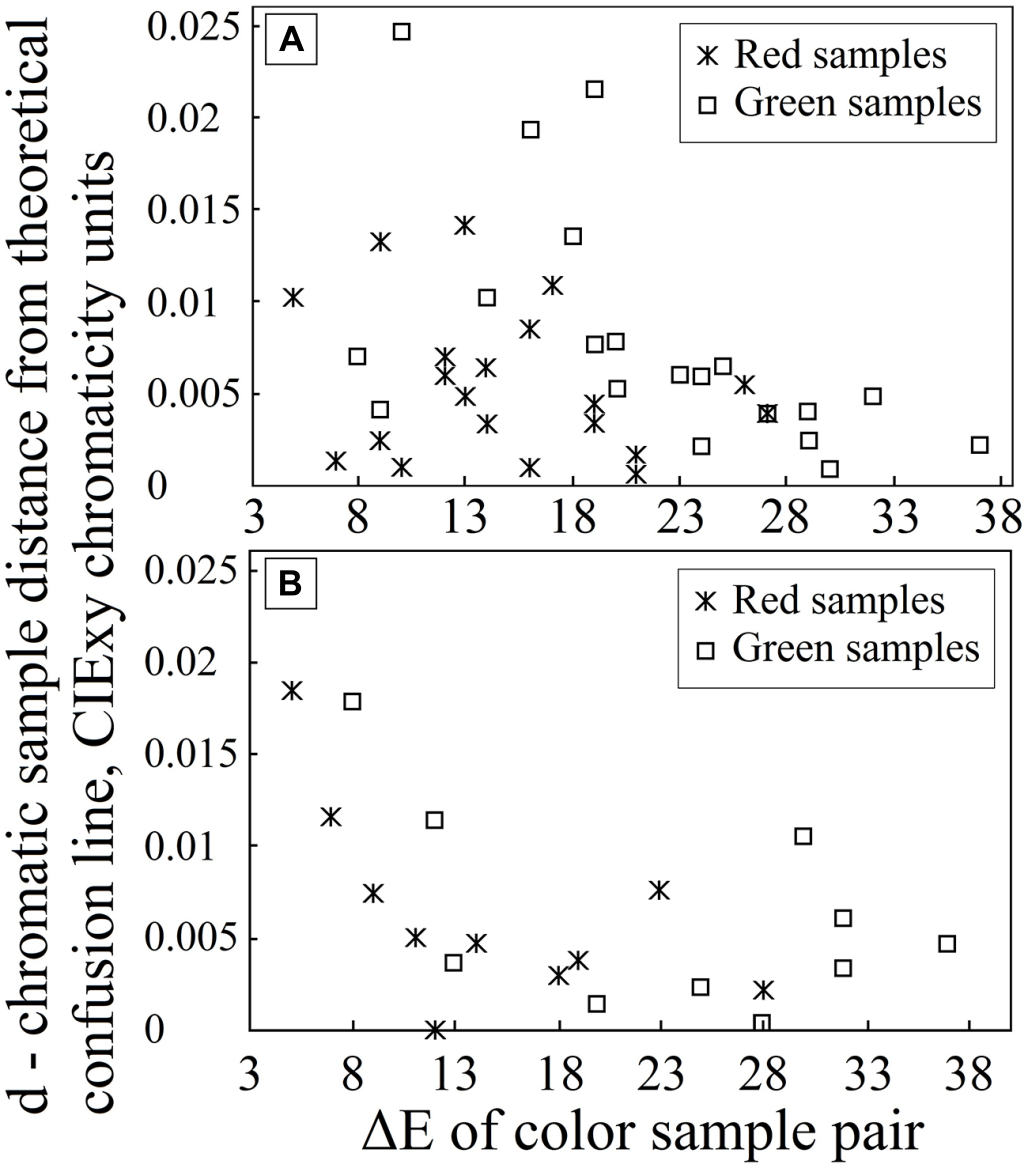
Graph **(A)** depicts the distance *d* for each chromatic sample from the line defined by the deutan confusion line copunctal point and the corresponding achromatic pair in the CIE *xy* color diagram (on the *y*-axis) as the sample color difference increases in CIE LAB color space (on the *x*-axis). Graph **(B)** depicts the corresponding distance *d* for protan plate analysis.

**FIGURE 4:**
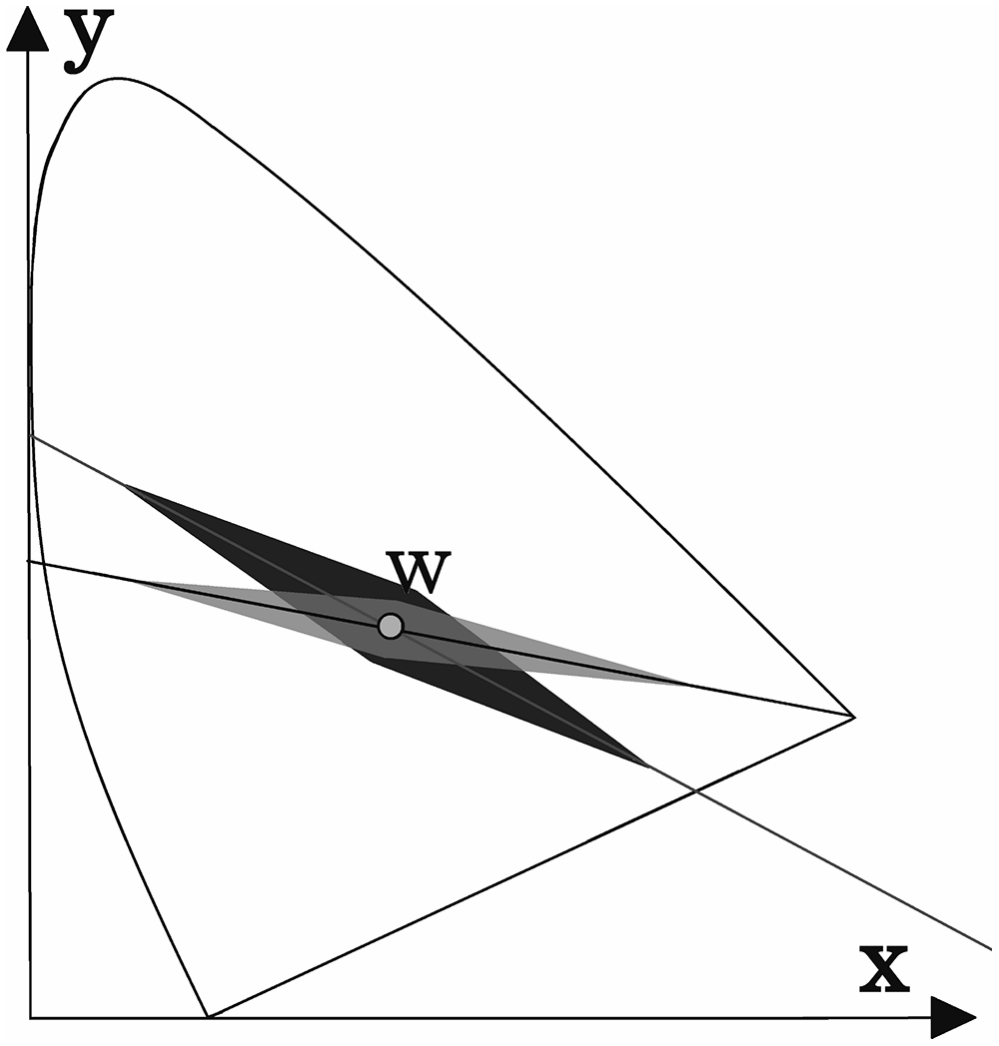
**A possible explanation for the results shown in Figure 3.** The acceptable shift (darker area) from the deutan confusion line decreases as the stimuli intensity increases. The acceptable shift distance for protans is shown as the lightest shaded area. The area suitable for screening purposes (i.e., the overlap area) is highlighted in gray. (These areas are not shown to scale.)

The results for deutan and protan sample color pairs are shown in **Figures 3A,B**, respectively. We find that larger shifts from the confusion line are acceptable for the observers if the sample was less saturated. As the color difference increased, the acceptable shift decreased for greenish and reddish samples. It is not surprising that larger shifts in the greenish zone of the CIE *xy* color diagram can be tolerated because of the size increment of the McAdam ellipses in the green area compared with the red area. We propose that the tolerated sample shifts for red–green color-vision-deficient individuals in the color diagram form the pattern shown in **Figure 4**. This hypothesis requires further investigation. Matching experiments for stimuli with a larger color difference might have been unsuccessful (even for deuteranope and protanope observers) because the color coordinate distribution of the printed areas exceeded the acceptable shifts in the saturated areas in the color diagram (see **Figure 4**).

### OBSERVERS

Children in two Latvian schools were tested to assess the clinical validity of the KAMS test. In total, 273 children (136 girls and 137 boys) from the Vainode region (Vainode secondary school) and the Priekule region (Gramzda elementary school) in Latvia participated. We chose these two schools in the periphery of our country to represent our population for a number of reasons: first, to join these schools, there are no specific requirements in terms of IQ, gender, or future choice of profession, which might lead to the sample being unrepresentative; second, school children form a group of individuals that is relatively homogeneous in age. The participation rate from both schools was 84.5%, and the age range was 7–19 years (with a mean age of 12.1 and a SD of 3.3 years). The study was conducted from mid-September to mid-October, 2012. Additionally, 57 volunteer individuals (with an age range of 7–67 years) with red–green deficiencies were examined from mid-September to mid-December, 2012. The study was conducted in accordance with the principles embodied in the Declaration of Helsinki Code of Ethics of the World Medical Association. All participants were unaware of the specific aims of the study.

### TESTING PROCEDURE

The room was illuminated using Narva LT-T8 18-W Colourlux plus CW (cool white) light bulbs. The color-rendering index of the bulbs was >80, the correlated color temperature was *T* = 4000 K, and the mean illumination of the test plates was 400 lx. It has been shown that fluorescent light sources are acceptable for color-vision testing (Dain et al., 1993).

The visual acuity of the children was examined using a LogMAR chart with the Landolt ring optotypes. If the near visual acuity was sufficient (better than 0.2 LogMAR), each child was tested using the fourth edition of the Richmond HRR plates (Richmond Products, Albuquerque, NM, USA), which is recommended for clinical use due to the high diagnostic accuracy (Cole et al., 2006; Dain, 2006; Cole, 2007), as well as the KAMS test. In the event that a single error was made in any of the above tests, an Oculus HMC (R) anomaloscope (type 47720) was used, and the Farnsworth D15 saturated and desaturated (Cole and Orenstein, 2003) testing procedure was carried out. Children were classified as color-vision-deficient only if they failed the anomaloscope test.

The HRR and KAMS tests were also carried out on the volunteer participants. If the anomaloscope and/or the D15 testing procedures were available, they were also performed. All of the volunteers undertook at least three of the above-mentioned tests. In total, results from 65 color-vision-deficient individuals (8 school children and 57 volunteers) were recorded; 21 subjects were classified as protans and 43 were classified as deutans [forming a large enough group of color-affected individuals for hypothesis testing, compared to other studies in the field (Huna-Baron et al., 2013; Lillo et al., 2014)]. It was not possible to interpret the results obtained with a single observer; hence, these results were excluded from further analysis.

### THE Δ*E* THRESHOLD

Following the diagnosis of each participant as deutan or protan, his or her results from the KAMS test were analyzed to determine the Δ*E* threshold for reddish and greenish plates separately. If, for example, the subject’s answers to the plates with Δ*E* values of 9, 10, 12, 17, 19, 21, and 27 were, respectively, wrong, wrong, wrong, wrong, correct, correct, and correct, then the Δ*E* threshold would be recorded as between 17 and 19 units (with a midpoint of 18 units and an uncertainty of ± 1 unit). If all plates were answered incorrectly, it was assumed that the Δ*E* threshold was between 27 and 60 units. At least two correct consecutive answers were required to assume that the threshold was between 0 and 27 units.

## RESULTS

### SPECIFICITY AND SENSITIVITY OF KAMS

The ability of the KAMS test to diagnose red–green color-vision deficiency was calculated based on data acquired in the population study (schools in Vainode and Gramzda). With the chosen criterion (> 1 error), the sensitivity of the test was 100%, and the specificity was 99.62%. The sensitivity was defined as the percentage of people with the condition that tested positive, and the specificity was defined as the percentage of people without the condition that tested negative (Norton et al., 2002).

### PROTAN/DEUTAN CLASSIFICATION AND DEFICIENCY SEVERITY LEVEL GRADING OF KAMS

**FIGURE 5:**
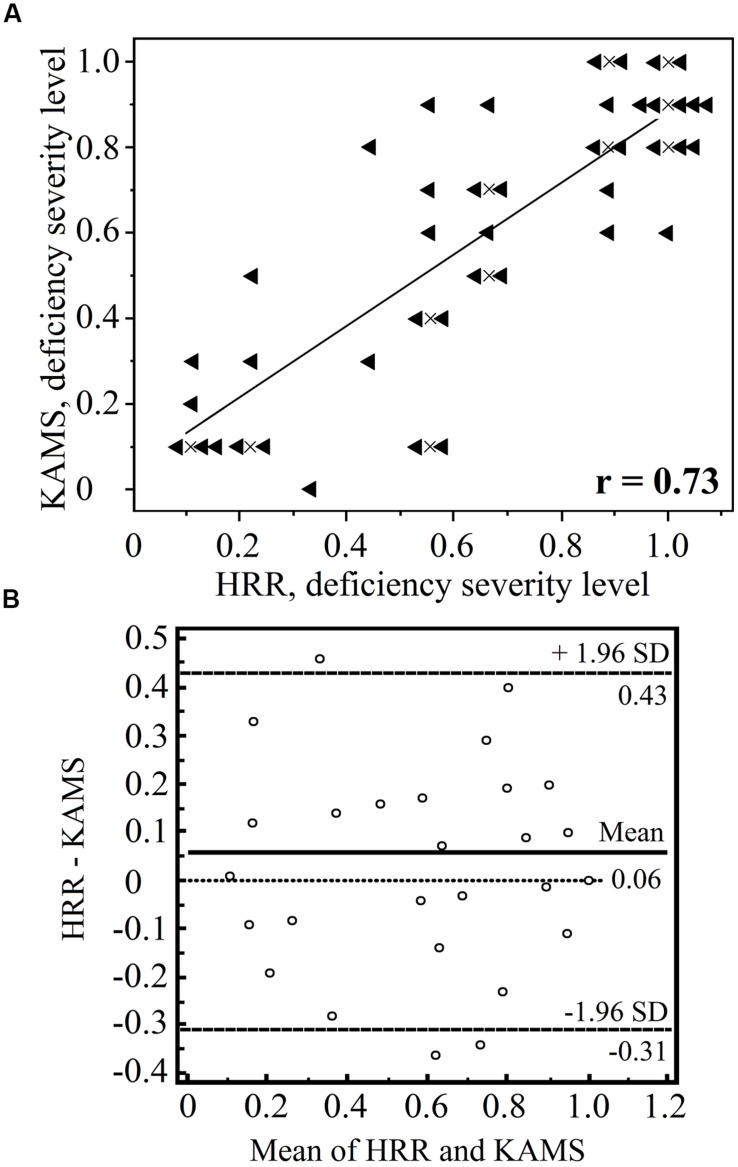
**Comparison of the Hardy–Rand–Rittler (HRR) and KAMS test results in terms of grading the severity of the deutan deficiency (*n* = 43).** The results of the screening plates are omitted. Graph **(A)** shows the number of errors made in the specific deutan grading plates with each test. A result of 1.0 corresponds to mistakes in all specific deutan plates, whereas a result of 0.5 means that 50% of the grading plates were determined correctly. The results of the two tests show a moderately high correlation (*r* = 0.73 and *p* < 0.001). A linear trend inferred from the data is also shown. Data points that overlap were offset from their true locations for demonstration purposes. The actual positions of the overlapping points are marked by the crosses. Graph **(B)** shows the Bland–Altman plot for both methods (using the MedCalc software package). No consistent bias was detected, and the difference in the sample means was not statistically significant (*p* = 0.05).

Although the PIC plate results alone should not be used to diagnose type of color-vision deficiency, we performed such an analysis to determine the suitability of the test for Δ*E* threshold determination for protans and deutans using separate plate groups. First, each individual participant was diagnosed as protan or deutan using the test battery described earlier. From the 64 individuals, in total, the HRR test misdiagnosed 1 deutan and 2 protans, and in seven cases, the HRR test failed to classify subjects (that is, it showed an equal number of errors in each column). The KAMS test misdiagnosed 5 deutans and 1 protan, and in two cases classification failed. We compared the ability of the KAMS test to grade the severity of the deficiency with that of the HRR test. Moderately good agreement was achieved (*r* = 0.74 and *p* < 0.01) in the case of protans. Test results for deutans are shown in detail in **Figure 5**.

### Δ*E* THRESHOLD VALUE IN CASE OF MILD DEFICIENCY AND CORRELATION WITH RGI

A convenient unit for characterizing red–green discrimination sensitivity is the red–green discrimination index (RGI), which is defined (Barbur et al., 2008) as

RGI=[1−(R−MR)÷73],

where *R* is the test subject’s Nagel matching range, and *MR* is the mean normal subject’s matching range. This definition results in RGI values that range from zero (in the case of dichromacy) to about one (in the case of high red–green color discrimination; Barbur et al., 2008).

**FIGURE 6:**
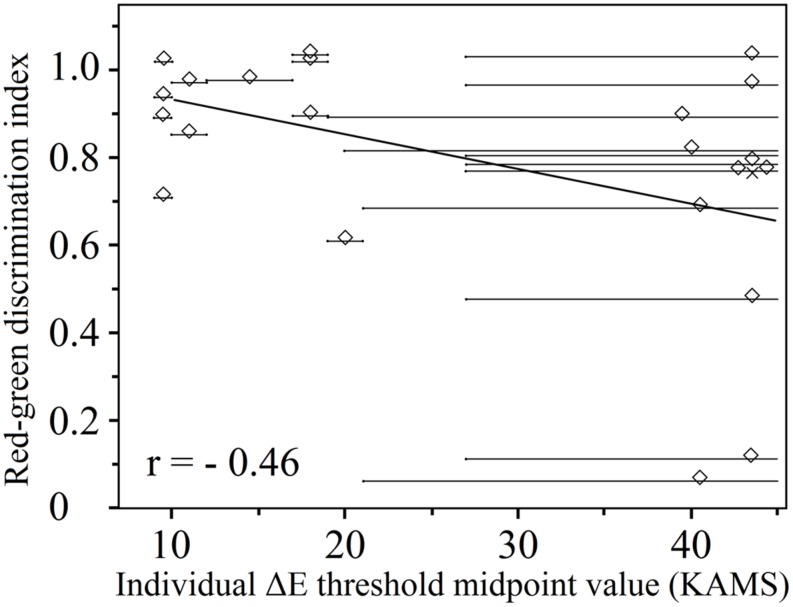
**Individual Δ*E* threshold midpoints obtained using the KAMS test as a function of the red–green discrimination index (deutan subjects, *n* = 22; 3 deuteranopes, 19 deuteranomalous).** Overlapping data points are offset from their true location for purposes of visualization. The actual positions of the overlapped points are marked by crosses. A linear regression to the data is also shown. Lighter gray horizontal lines show the uncertainties in the thresholds.

As expected, there was a moderately strong negative correlation between the midpoint threshold values obtained using the KAMS test in deutan (*n* = 22) observers (*r* = -0.46 and *p* = 0.042 for thresholds obtained with reddish plates, and *r* = -0.44 and *p* = 0.042 for thresholds obtained using greenish plates). The relationship is shown in **Figure 6** in the case of deutan observers for thresholds obtained by reddish plates. Identical (positive only) correlation coefficients and *p*-values were obtained for the correlation of the anomaloscope matching range to the obtained threshold values. Because of the small number of protan observers (*n* = 13), a negligible correlation was obtained for threshold values obtained using the green test plates (*r* = -0.09 and *p* = 0.775), and a moderate negative correlation was obtained for threshold values from the reddish test plate results (*r*= -0.33 and *p* = 0.263).

**Figure 6** shows individual Δ*E* threshold midpoints; two data distributions can be distinguished, which were separated by an empty area in the center of the plot. In reality, it was possible to obtain a valid threshold region with limits from both sides only for the participants corresponding to data points on the left side of this graph. For the participants marked by points on the right, it was possible (with the PIC plates available) to be certain only about the smallest threshold midpoint region. To tackle this problem, a Δ*E* value of 60 units was used as the largest possible threshold value in the case of each participant for the data clustered on the right-hand side of the graph. The midpoint was calculated keeping in mind that there is a large position uncertainty in the precise thresholds of the midpoints. In other words, those subjects with lower Δ*E* discrimination thresholds had a higher red–green discrimination index; however, subjects with higher Δ*E* discrimination thresholds than we could determine may have various RGIs. We can only suggest that, based on the distribution of data points on the left-hand side of the figure, these other data points might be aligned along a line with a negative gradient (as shown in **Figure 6**), if the testing method were improved. Nonetheless, we have shown that a lower Δ*E* discrimination threshold is consistent with smaller matching ranges in the anomaloscope testing procedure for deuteranomalous observers.

Color vision is known to change with age (Roy et al., 1991). The group of individuals with affected color vision that we studied was not coherent in terms of age. We carried out separate data analysis to eliminate the possible effects of age on the variation of the acquired KAMS thresholds (as well as the anomaloscope matching range midpoint values). A weak correlation was found, ruling out direct causation of threshold variance due to age differences (charts attached to the supplementary material).

## DISCUSSION

We have compared the KAMS test with the Richmond HRR test throughout this study because the latter is one of the best clinical PIC tests (Cole et al., 2006; Dain, 2006; Cole, 2007). The high sensitivity and specificity of KAMS suggests that although the KAMS test was created for scientific (as opposed to clinical) purposes, it is a potential supplementary test for use in both research and clinics; we do not suggest replacing currently used tests with the KAMS test. The good level of agreement in terms of the grading results obtained using KAMS and the HRR test suggests that the tests are comparable; however, our plate set has scope for use in psychophysical investigations. The main novelty of our research is the depiction of the results of a PIC test via a discrimination threshold described by a numerical value. The total color difference is a commonly used quantity in science. The results given in terms of total color difference may be useful and easy to interpret for specific job applications or for product developers (the results depict color-sensitivity loss in comparison with the color-discrimination ability of a normal observer for neutral colors). Using a limited number of plates (five for greenish and nine for reddish directions in the CIE *xy* coordinates), we were able to determine individual Δ*E* discrimination thresholds for deutans. Standardization and development of finer plate grading might improve the reliability of the results; for example, by reducing the uncertainty interval in the data (see **Figure 6**). By improving the test method, we could clarify whether individual thresholds are fixed or variable, as well as the amount of variability.

Our results show a correlation of the individual Δ*E* thresholds and the RGI for deuteranomalous individuals. Such an outcome was expected, as Cole (2007) reported similar results for HRR grading and the Nagel matching range (*r* = 0.58). However, the Nagel matching range is not correlated with the obtained red–green thresholds in the Color Assessment and Diagnosis test, which uses neutral colors for the background, similar to the HRR and KAMS test (Barbur et al., 2008). These results might be explained by differences in the perception of rapidly moving and changing images compared with that of static images. However, the following question remains: why are the results of the Farnsworth D15 test not comparable to the anomaloscope data (Birch, 2008)? A different approach to the analysis of the experimental data might provide other results. Furthermore, our results might be partly affected by the effect of normal age-related changes in color vision. A larger and more age-coherent sample size for the color-vision impaired group (especially in case of the protan observers) would be necessary to make a full assessment of the effects analyzed in this study.

## CONCLUSION

(1) The KAMS test was found to be valid for the screening of congenital red–green color-vision deficiencies, and the sensitivity and specificity are comparable to those of the HRR test (2002, 4th edition).

(2) The design of psychophysical tests can be used to assess red–green color-vision deficiencies and obtain individual color saturation discrimination thresholds in the case of anomalous trichromates.

(3) The size of the anomaloscope-matching range in Nagel units for deutan observers exhibited a moderately strong positive correlation (a moderately strong negative correlation, in the case of RGI) with the acquired Δ*E* threshold midpoints of the psychophysical testing procedure used in this study.

## Conflict of Interest Statement

The authors declare that the research was conducted in the absence of any commercial or financial relationships that could be construed as a potential conflict of interest.

## References

[B1] Baraas R. C. Foster D. H. Amano L. Nascimento S. M. C. (2010). Color constancy of red-green dichromats and anomalous trichromats. *Invest. Ophthalmol. Vis. Sci.* 51 2286–2293 10.1167/iovs.09-4576 19892868 PMC2868405

[B2] Barbur J. L. Rodrigues-Carmona M. Harlow J. A. Mancuso K. Neitz J. Neitz M. (2008). A study of unusual Rayleigh matches in deutan deficiency. *Vis. Neurosci.* 25 507–516 10.1017/S0952523808080619 18598426 PMC3044924

[B3] Berns R. S. (2006). *Billmeyer and Saltzman’s Principles of Color Technology* 3rd Edn. New York, NY: John Wiley & Sons.

[B4] Birch J. (1993). *Diagnosis of Defective Colour Vision.* New York, NY: Oxford University Press.

[B5] Birch J. (2008). Failure of concordance of the Farnsworth D15 test and the Nagel anomaloscope matching range in anomalous trichromatism. *Vis. Neurosci.* 25 451–453 10.1017/S0952523808080231 18598417

[B6] Cole B. L. (2007). Assessment of inherited colour vision defects in clinical practice. *Clin. Exp. Optom.* 90 157–175 10.1111/j.1444-0938.2007.00135.x 17425762

[B7] Cole B. L. Lian K. Y. Lakkis C. (2006). The new Richmond HRR pseudoisochromatic test for colour vision is better than the Ishihara test. *Clin. Exp. Optom.* 89 73–80 10.1111/j.1444-0938.2006.00015.x 16494609

[B8] Cole B. L. Orenstein J. M. (2003). Does the Farnsworth D15 test predict the ability to name colours? *Clin. Exp. Optom.* 86 221–229 10.1111/j.1444-0938.2003.tb03109.x 12859240

[B9] Dain S. J. (2004). Colorimetric analysis of four editions of the Hardy-Rand-Rittler pseudoisochromatic tests. *Vis. Neurosci.* 21 437–443 10.1017/S0952523804213475 15518226

[B10] Dain S. J. (2006). Illuminant and observer metamersim and the Hardy-Rand-Rittler color vision test editions. *Vis. Neurosci.* 23 685–694 10.1017/S095252380623356X 16998976

[B11] Dain S. J. Honson V. J. Curtis C. T. (1993). Suitability of fluorescent tube light sources for the Ishihara test as determined by colorimetric methods. *Colour Vis. Defic.* 56 327–333 10.1007/978-94-011-1856-9_33

[B12] Fomins S. Ozolinsh M. (2011). Multispectral analysis of color vision deficiency tests. *Mater. Sci. (Medžiagotyra)* 17 1392–1320 10.5755/j01.ms.17.1.259

[B13] Huna-Baron R. Glovinsky Y. Habot-Wilner Z. (2013). Comparison between Hardy-Rand-Rittler 4th edition and Ishihara color plate tests for detection of dyschromatopsia in optic neuropathy. *Graefes Arch. Clin. Exp. Ophthalmol.* 251 585–589 10.1007/s00417-012-2073-x 22688625

[B14] Lee D. Y. (2006). Color changes in the red-green plates of the 50-year-old AO HRR color vision test. *Vis. Neurosci.* 23 681–684 10.1017/S0952523806233054 16962013

[B15] Lee D. Y. Honson M. (2003). Chromatic variation of Ishihara diagnostic plates. *Color Res. Appl.* 28 267–276 10.1002/col.10161

[B16] Lillo J. Alvaro L. Moreira H. (2014). An experimental method for the assessment of color simulation tools. *J. Vis.* 14 15 10.1167/14.8.15 25052695

[B17] Luse K. Fomins S. Ozolinsh M. (2012). Pseudoisochromatic test plate colour representation dependence on printing technology. *IOP Conf. Ser. Mater. Sci. Eng.* 38 012024 10.1088/1757-899X/38/1/012024

[B18] Luse K. Ozolinsh M. Fomins S. Gutmane A. (2013). Multispectral analysis and cone signal modeling of pseudoisochromatic test plates. *IOP Conf. Ser. Mater. Sci. Eng.* 49 012041 10.1088/1757-899X/49/1/012041

[B19] Norton T. T. Corliss D. A. Bailey J. E. (2002). *The Psychophysical Measurement of Visual Function.* Burlington, MA: Butterworth–Heinemann.

[B20] Roy M. S. Podgor M. J. Collier B. Gunkel R. D. (1991). Color vision and age in normal North American population. *Graefes Arch. Clin. Exp. Opthalmol.* 229 139–144 10.1007/BF00170545 2044973

[B21] Smith V. C. Pokorny J. Yeh T. (1993). Pigment tests evaluated by a model of chromatic discrimination. *J. Opt. Soc. Am. A Opt. Image Sci. Vis.* 10 1773–1784 10.1364/JOSAA.10.001773 8350160

[B22] Wenzel K. Samu K. (2012). Pseudo-isochromatic plates to measure colour discrimination. *Acta Polytechnica Hungarica* 9 1–11.

