# Supplementary material for: Color-discrimination threshold determination using pseudoisochromatic test plates
Source: Front Psychol. 2014 Nov 27;5:1376. doi: 10.3389/fpsyg.2014.01376 (PMC4245893; doi:10.3389/fpsyg.2014.01376)
Supplement: Supplementary file 1 [file Data_Sheet_1.DOCX]

We carried out separate data analysis to eliminate the possible effects of age on the variation of the anomaloscope matching range midpoint values (see Graph 1) (as well as the acquired KAMS thresholds (Graph 2)). The acquired correlation levels were weak, ruling out direct causation of threshold variance due to age differences.


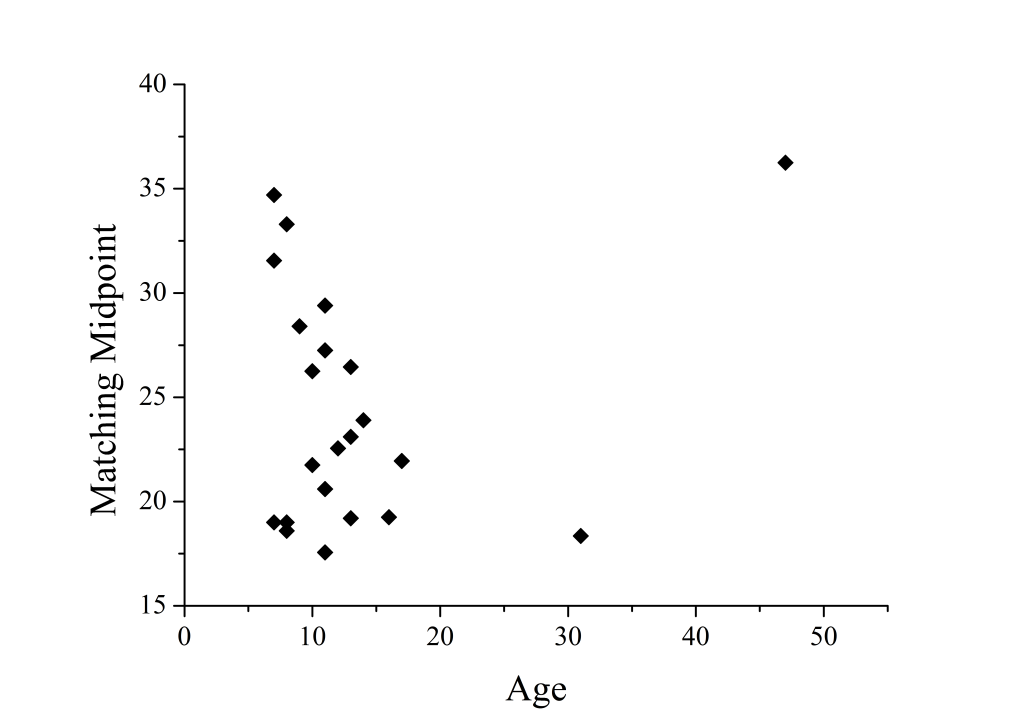


Graph 1. The Matching Midpoint value dependence on deutan observer age is shown (*r* = 0.18).


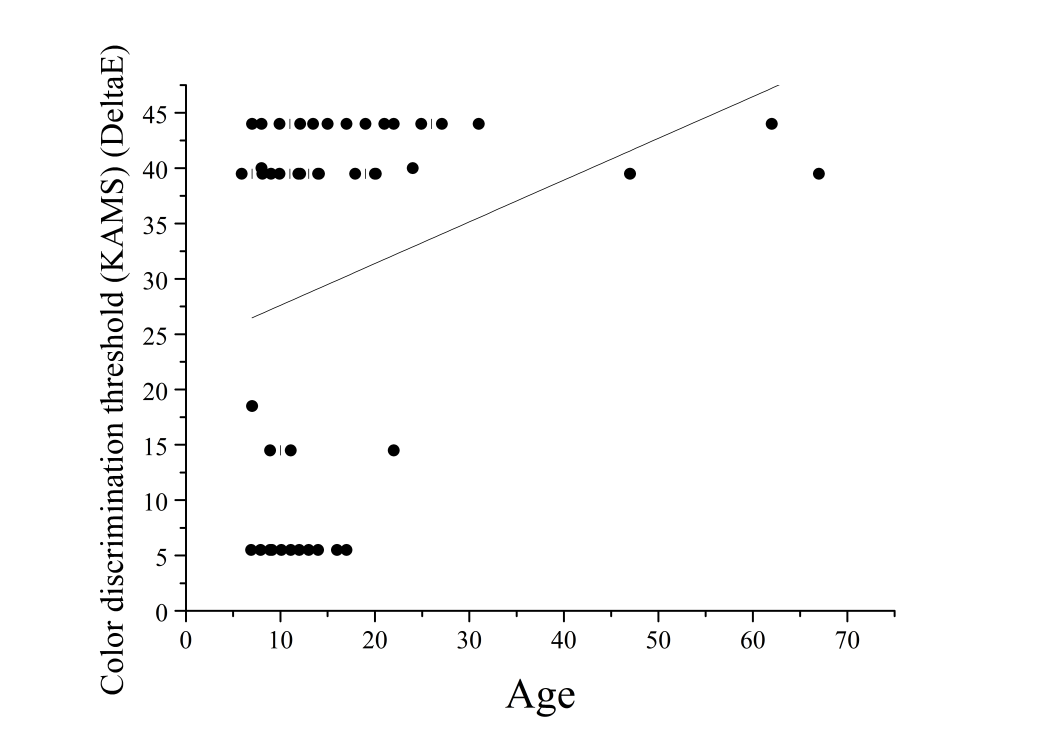


Graph 2. KAMS threshold value (in *ΔE* units) dependence on deutan observer age is shown (*r* = 0.19). Overlapping data points are offset from their true location for purposes of visualization. The actual positions of the overlapped points are marked by vertical lines.
